# Point-of-Care Ultrasound (POCUS)-Guided Management of Cardiogenic Shock in COVID-19 Fulminant Myocarditis With Combined Veno-Arterial Extracorporeal Membrane Oxygenation and Impella (ECPELLA): A Case Report

**DOI:** 10.7759/cureus.60665

**Published:** 2024-05-20

**Authors:** Shunichi Kato, Eiji Kurosaka, Kentaro Nakata

**Affiliations:** 1 Department of Cardiology, Saitama Red Cross Hospital, Saitama, JPN; 2 Advanced Emergency and Critical Care Center, Saitama Red Cross Hospital, Saitama, JPN

**Keywords:** vexus, tapse, impella, ecpella, va-ecmo, covid 19, fulminant myocarditis, point-of-care-ultrasound

## Abstract

The COVID-19 pandemic, which has been raging globally, has been reported to cause not only pneumonia but also various cardiovascular diseases. In particular, myocarditis poses a serious risk if it becomes severe. As a characteristic of myocardial damage in this disease, right ventricular dysfunction is frequently reported, and biventricular failure is not uncommon. In cases where cardiogenic shock occurs, ECPELLA, which combines veno-arterial extracorporeal membrane oxygenation and Impella, is used for management. Currently, in Japan, ECPELLA is the central treatment for severe biventricular failure in the acute phase. However, its management method has not been established. Weaning from ECPELLA requires the following three conditions: (1) improvement of left ventricular function; (2) improvement of right ventricular function; and (3) optimization of circulating plasma volume. However, since these conditions change moment by moment, frequent and detailed assessments are necessary. Nevertheless, considering the need for isolation due to COVID-19, there are limitations on the tests that can be performed. In this regard, point-of-care ultrasound (POCUS) allows repeated bedside evaluations while maintaining infection protection. We report that in the case of severe COVID-19-related myocarditis, the use of POCUS enabled the preservation of cardiac function and appropriate timing for weaning from ECPELLA.

## Introduction

The global COVID-19 pandemic continues, but the number of infections and deaths is decreasing. COVID-19 not only causes pneumonia but also various cardiovascular diseases, especially myocarditis, which carries a risk of becoming severe [1]. In Japan, the combination of veno-arterial extracorporeal membrane oxygenation (VA-ECMO) and Impella, called “ECPELLA,” is proposed in the guidelines for managing biventricular failure due to fulminant myocarditis [2]. However, there are few established reports on its management method.

We report a case of biventricular failure due to COVID-19-related fulminant myocarditis that required ECPELLA, where point-of-care ultrasound (POCUS) played a crucial role in management. POCUS is an important noninvasive bedside echocardiographic examination that can be repeatedly performed, making it essential for cardiovascular intensive care where the patient’s condition changes rapidly.

## Case presentation

**Figure 1:**
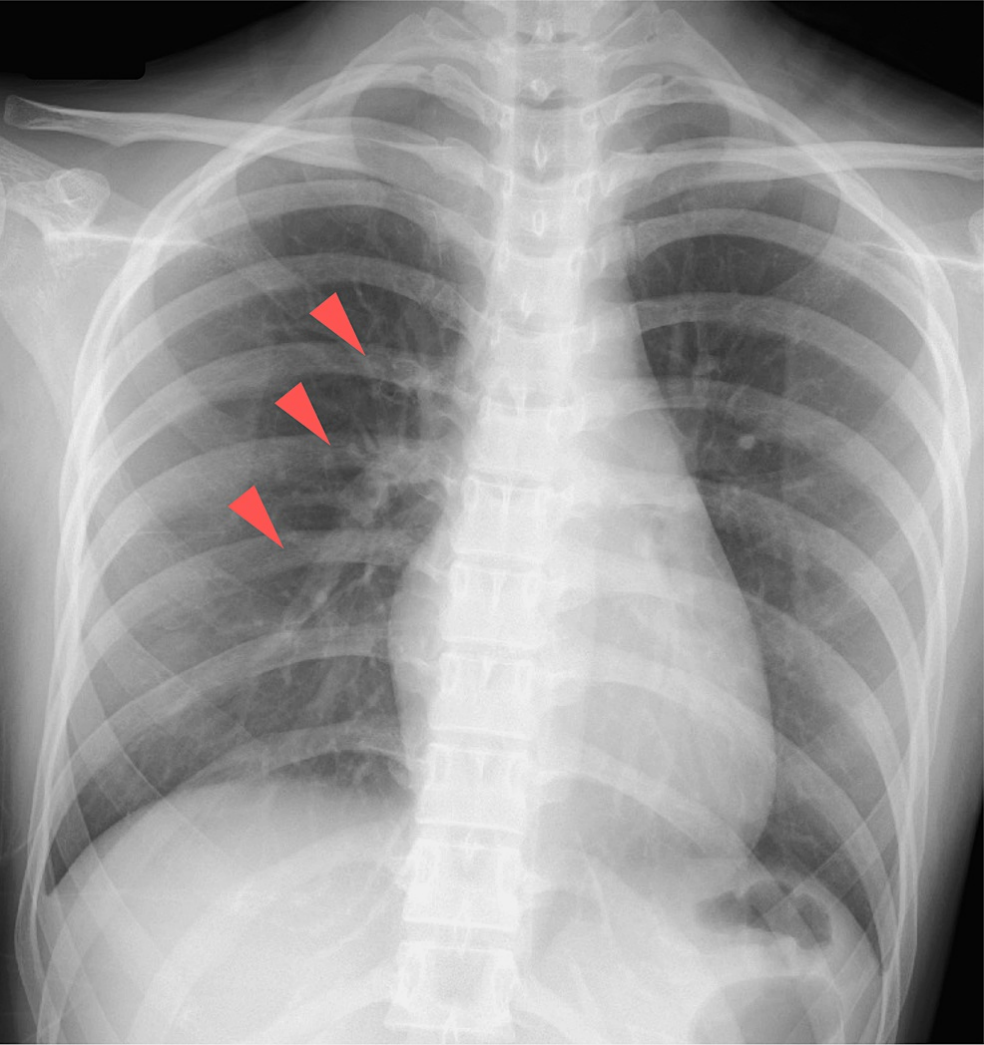
Chest X-ray on admission The chest X-ray showed a slight enhancement of hilar shadow (red arrowheads), no consolidation suggestive of pneumonia, and the cardiothoracic ratio was 45.6%.

**Figure 2:**
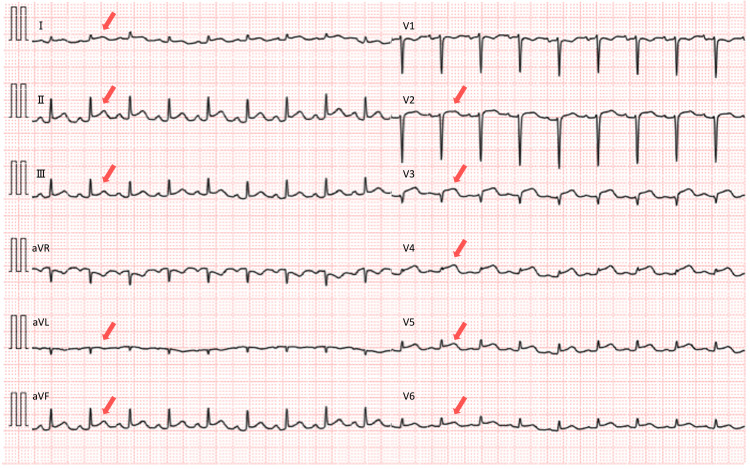
12-lead electrocardiogram on admission The 12-lead electrocardiogram showed ST-segment elevation in multiple leads (red arrows).

The patient was a 23-year-old woman. Five days before admission, she had a fever of 38 °C and tested positive for COVID-19 antigen, leading to home isolation. Three days before admission, she developed chest pain, which initially improved with analgesics but later recurred with dyspnea, prompting emergency transport. She had no past medical history, family history, allergies, or smoking history. She was unvaccinated against COVID-19. On admission, her blood pressure was 88/64 mmHg, her heart rate was 120 bpm, and she was in shock with peripheral coldness. The chest X-ray (Figure 1) showed a slight enhancement of hilar shadow (red arrowheads), no pneumonia, and a cardiothoracic ratio of 45.6%. The 12-lead electrocardiogram showed ST-segment elevation in multiple leads (Figure 2). Blood tests revealed high-sensitivity troponin I at 6,173.5 pg/ml, brain natriuretic peptide at 754.7 pg/ml, creatine kinase at 1,725 U/L, and lactate at 5.97 mmol/L. Transthoracic echocardiography showed an ejection fraction of around 20%, diffuse severe hypokinesis, wall thickening as edematous, and pericardial effusion. Based on these findings, she was diagnosed with COVID-19-related fulminant myocarditis, cardiogenic shock, and the Society for Cardiovascular Angiography & Interventions (SCAI) cardiogenic shock classification C [3].

Subsequently, the patient underwent intubation in the hybrid emergency room and coronary angiography, which revealed no significant stenosis, and Impella CP (Abiomed, Inc., Danvers, Massachusetts, United States) was inserted. She was admitted to the ICU and administered the standard dose of dexamethasone and remdesivir for COVID-19. However, her lactate level continued to rise, reaching 10.89 mmol/L, and her condition was classified as SCAI cardiogenic classification D. VA-ECMO was added, and ECPELLA management was initiated. Due to an inflammatory sepsis-like condition and bleeding from the VA-ECMO insertion site, massive transfusion and fluid resuscitation were required, stabilizing her hemodynamics on Day 2. On Day 3, diuresis started to wean off mechanical circulatory support, as her weight had increased by 20 kg since admission. During VA-ECMO weaning, tricuspid annular plane systolic excursion (TAPSE) and tricuspid valve systolic wall motion velocity (TV-S’) were monitored to assess right ventricular function, which showed no worsening. Simultaneously, the Venous Excess Ultrasound (VExUS) scoring was used to evaluate fluid tolerance, and Grade 2 was observed on Day 4, prompting increased diuresis up to 5,105 ml/day, leading to improvement. With this management, successful weaning from VA-ECMO and Impella CP was achieved on Day 5. The patient was extubated on Day 9 and discharged from the ICU on Day 12. She was discharged home on Day 21.

## Discussion

COVID-19-related myocarditis occurs in 0.2% of hospitalized patients. Some cases become severe, with 20% requiring mechanical circulatory support, and the mortality rate in those cases reaches 6.6% [4]. The proposed mechanism for myocarditis is that SARS-CoV-2 directly infects cardiomyocytes via the angiotensin-converting enzyme 2 receptor, causing myocarditis [5]. Previous studies reported that adult patients infected with COVID-19 could develop a multisystem inflammatory syndrome (MIS-A), including fulminant myocarditis. To elaborate further, it has been noted that COVID-19-associated myocarditis may be more severe if it does not meet strict MIS-A criteria [6]. A case of non-MIS-A myocarditis requiring MCS has been reported, and the article states that it is important to distinguish between these two phenotypes [7]. However, these have not yet been reported, and further studies are needed.

While cardiac MRI (CMR) is useful for diagnosing COVID-19-related myocarditis [8], performing CMR during the peak of infection is challenging. Therefore, bedside echocardiographic evaluation becomes crucial. The American Society of Echocardiography (ASE) has issued guidelines for protective measures during bedside echocardiography in COVID-19 patients [9]. Previous reports indicate that right ventricular dysfunction is the most common initial abnormality in COVID-19-related myocarditis [10,11]. In recent years, there have been reports of using Impella CP for the treatment of fulminant myocarditis. However, Impella CP support is limited in cases of right ventricular failure. Because Impella CP assists hemodynamics by drawing blood from the left ventricle to the aorta, it cannot assist with the loss of autologous right heart function. While right ventricular Impella devices are available abroad, the use of two Impella called the “BiPella approach,” which can assist each of the left and right ventricles, has been reported to treat COVID-19-related fulminant myocarditis [12]. However, they are not approved in Japan as of 2024. Therefore, the combination of VA-ECMO and Impella, known as “ECPELLA,” becomes the primary treatment for severe biventricular failure in Japan. The concept of ECPELLA is that VA-ECMO bypasses blood from the right atrium to the aorta, then the afterload is canceled by Impella, preventing an increase in left ventricular end-diastolic pressure. The previous report described the prognosis as favorable if adequate hemodynamic support is provided [13].

**Figure 3:**
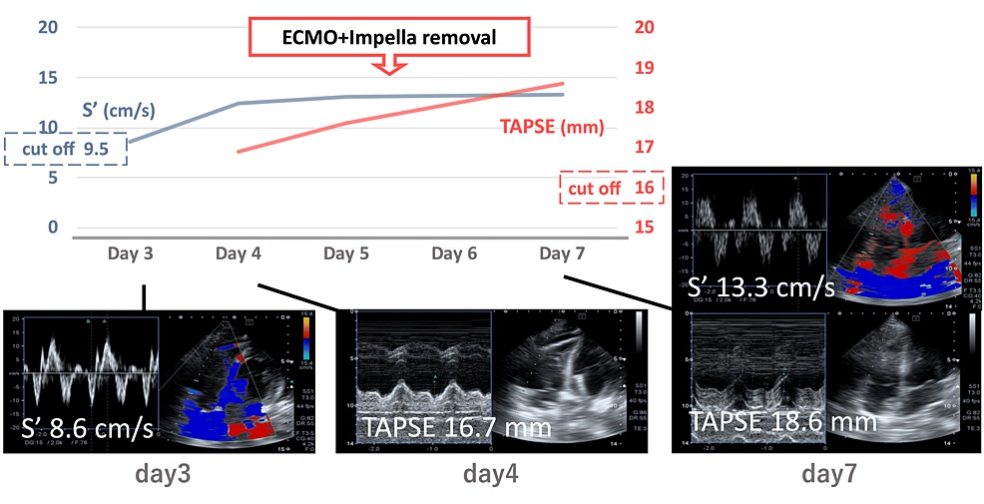
The course of right heart function measured by POCUS TAPSE gradually recovered (red line, 16.7 mm on Day 4, 17.6 mm on Day 5, and 18.6 mm on Day 7). TV-S’ also gradually recovered (blue line, 8.6 cm/s on Day 3, 12.4 cm/s on Day 4, 13.1 cm/s on Day 5, and 13.3 cm/s on Day 7). POCUS, point-of-care ultrasound; TAPSE, tricuspid annular plane systolic excursion; TV-S’, tricuspid valve systolic wall motion velocity

In Impella-assisted circulatory management, the pulmonary artery pulsatility index (PAPi) is a useful parameter, with values below 0.9 associated with right ventricular dysfunction [14]. The effectiveness of PAPi has also been reported for implantable ventricular assist devices [15]. However, with concomitant VA-ECMO, the right atrial drainage and preload reduction may lower the pulmonary artery pressure and cardiac output [16], potentially affecting the utility of PAPi. Therefore, we monitored right heart function with multiple parameters by using not only PAPi but also TAPSE and TV-S’ measured by POCUS in this case. PAPi gradually recovered from 0.05 on Day 1 to 1.3 on Day 7 and correlated with TAPSE and TV-S’. Weaning from ECPELLA requires the following three conditions: (1) improvement of left ventricular function; (2) improvement of right ventricular function; and (3) optimization of circulating plasma volume. In this case, we monitored right ventricular function using POCUS during VA-ECMO weaning. Specifically, we measured TAPSE and TV-S’ daily to assess for worsening right ventricular failure. We confirmed the optimization of circulating plasma volume by diuresis, so we reduced ECMO flow by about 0.5 L/min/day and also added Impella CP with gradual weaning. Each time, we reevaluated using POCUS and adjusted the diuretic dose. The results showed a gradual improvement in TAPSE and TV-S’, with no recurrence of abnormalities, allowing smooth weaning (Figure 3).

**Figure 4:**
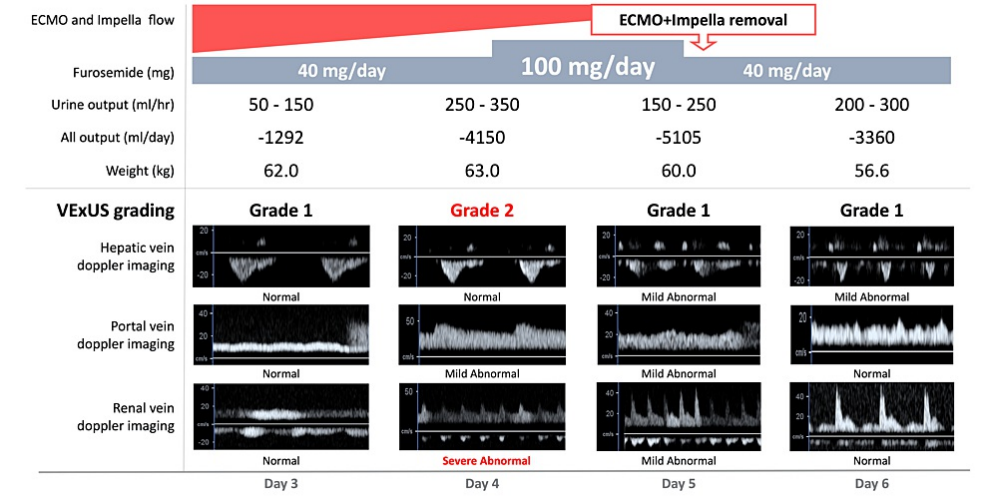
The course of the fluid tolerance with VExUS grading measured by POCUS POCUS, point-of-care ultrasound; VExUS, Venous Excess Ultrasound

Additionally, we optimized circulating plasma volume using POCUS during VA-ECMO weaning. Due to massive fluid resuscitation and transfusion from admission, the patient’s weight had increased by 20 kg by Day 3. The VExUS method, which scores and grades fluid tolerance using POCUS, has been recently reported for evaluating fluid tolerance [17]. Previous studies in cardiovascular surgery patients showed that VExUS Grades 2 and 3 had positive likelihood ratios of 2.86 and 6.37, respectively, for acute kidney injury compared to Grade 1, with better predictive accuracy than elevated central venous pressure [18]. On Day 4, the VExUS grade increased to 2, likely due to the VA-ECMO weaning. Consequently, the furosemide infusion was increased to 100 mg/day, resulting in a urine output of 5,105 ml/day and improvement to Grade 1 (Figure 4).

Previous reviews have stated that pulmonary artery catheters are an important monitoring tool but are not routinely used due to risks such as invasion and infection. Static measures obtained from pulmonary artery catheters alone may not be sufficient to assess circulating plasma volume. In this regard, POCUS is noninvasive, repeatable, and has no risk of infection. It is also possible to evaluate excess circulating plasma volume by interpreting Doppler waveforms in the veins of organs to identify changes in waveforms due to congestion [19]. However, due to the limitation of this article as a single case experience, we emphasize the need for larger studies to validate the role of POCUS in managing fulminant myocarditis with ECPELLA support.

## Conclusions

In COVID-19 infection, myocarditis can occur in addition to lung involvement. In cases of fulminant myocarditis leading to biventricular failure, mechanical circulatory support with ECPELLA may be required. In such cases, monitoring not only left ventricular function but also right ventricular function and fluid tolerance using POCUS is crucial. By effectively utilizing these parameters, appropriate adjustment of mechanical support and controlled weaning can be achieved, leading to favorable outcomes.
